# Flow Stress Model of Hot Deformation for CoNiV Medium Entropy Alloy

**DOI:** 10.3390/ma19132894

**Published:** 2026-07-06

**Authors:** Qixuan Hao, Biao Zhang, Yuntian Du, Yaliang Liu, Minghe Zhang, Yunli Feng

**Affiliations:** 1 College of Metallurgy and Energy, North China University of Science and Technology, Tangshan 063210, China; 17732394606@163.com (Q.H.); bzhang187@163.com (B.Z.); liuyaliang@ncst.edu.cn (Y.L.); tsfengyl@163.com (Y.F.); 2 College of Science, North China University of Science and Technology, Tangshan 063210, China; dyt2585526816@163.com

**Keywords:** hot deformation, Zerilli-Armstrong model, machine learning, CoNiV MEA

## Abstract

Hot compression experiments were performed to characterize the high-temperature deformation behavior of CoNiV medium-entropy alloy (MEA). Hot compression tests were carried out using a Gleeble-3500 thermomechanical simulator over strain rates of 0.001 s^−1^ to 1 s^−1^ and temperatures ranging between 950 °C and 1100 °C. Based on the experimentally determined hot compression data, three models for predicting the flow stress of CoNiV MEA were established: the Zerilli-Armstrong (Z-A) constitutive model, an artificial neural network (ANN) model, and a gated recurrent unit (GRU) model. This study comprehensively evaluated the prediction accuracy of each model using the coefficient of determination (R^2^), mean absolute error (MAE), and root mean square error (RMSE). The results show that, compared with the other two models, the Z-A model cannot accurately predict the flow behavior of CoNiV MEA in the studied hot-working regime. The R^2^ value of the ANN model is 0.98974, while the GRU model exhibits the highest predictive capability, with an R^2^ value of 0.98981, an MAE of 6.29621, and an RMSE of 13.10832. The proposed model demonstrates superior prediction accuracy compared with other models, enabling high-precision characterization of the high-temperature evolution of the flow stress in the CoNiV MEA. This study provides a theoretical foundation for the design and optimization of hot working parameters for the CoNiV MEA.

## 1. Introduction

Unlike traditional alloys, MEAs adopt an equiatomic or near equiatomic mixture of multiple principal elements, typically forming solid solution phases with complex compositions [1,2,3]. MEAs have drawn significant attention on account of their excellent performances and unique structures [4,5,6,7]. As a classic representative of face-centered cubic (FCC) structured MEAs, the CoCrNi alloy features concurrent strengthening and ductilization at cryogenic temperatures [8]. At room temperature, the CoCrNi alloy achieves a yield strength (YS) of 300 MPa and an elongation of 68% [9]. Under cryogenic conditions (77 K), the CoCrNi alloy possesses more outstanding mechanical properties, with a YS of 657 MPa and an elongation after fracture of 90% [8]. Sohn et al. [10] substituted Cr atoms with V atoms to improve the room temperature yield strength of FCC-structured MEAs. Owing to the marked discrepancy in atomic radii between V and the other two species, the crystal lattice experiences substantial distortion. By regulating the grain size, the room temperature YS approached 1 GPa while retaining favorable ductility. Such exceptional properties have rendered CoNiV alloy a research hotspot in the field of MEAs. The preparation of CoNiV MEAs involves processes such as melting, casting, cold rolling, and heat treatment. Casting-induced porosities and coarse columnar grains significantly deteriorate the mechanical response of as-cast alloys; consequently, such materials fail to meet the basic requirements for direct service in engineering components [11,12]. To refine the microstructure of as-cast alloys and achieve better mechanical properties, heat treatment is an effective method. A significant gap still exists in the understanding of hot deformation behaviors for CoNiV alloys, despite the wealth of studies dedicated to their mechanical properties at room temperature.

Thoroughly investigating the hot deformation behavior of a material enables us to formulate scientifically sound hot working parameters. Constitutive models serve to quantitatively describe the deformation characteristics of materials when subjected to coupled multi-physical fields and external mechanical loading. By establishing constitutive relationships among key variables such as stress, strain, strain rate, and temperature, these models predict the mechanical response of materials under complex conditions like hot working, thereby providing a data foundation for analyzing deformation behavior, designing processing windows, and optimizing processing techniques. Using the Z-A model as a predictive tool, Han et al. [13] evaluated the high-temperature flow stress of Fe_49_Mn_3.2_Cr_9.6_Co_8.2_ HEA as a function of strain rate and temperature. A BKA-SVR machine learning model, constructed by optimizing traditional support vector regression (SVR) with the Black Kite Algorithm (BKA), was developed by Hu et al. [14] and applied to the prediction of flow stress. In small-sample prediction scenarios, the BKA-SVR model delivered highly accurate flow stress predictions, achieving an R^2^ of 0.99527 and an MAE of 6.0667. Although the SVR model exhibits strong generalization capability in small-sample data scenarios, it cannot handle dynamic evolution processes.

Leveraging its inherent nonlinear mapping and self-adaptive learning mechanisms, the artificial neural network (ANN) model excels at deciphering the intricate high-dimensional relationships between flow stress and the multivariable parameters during hot deformation [15,16]. This model does not require pre-assuming the form of the constitutive equation; it achieves high-precision stress prediction solely through data-driven methods, demonstrating generalization ability and adaptability, making it particularly suitable for processing multivariate and nonlinear hot deformation data. For the purpose of forecasting the flow behavior of TA31 alloy at elevated temperatures, Hu et al. [17] established a back-propagation ANN model. The constructed model exhibited a high correlation coefficient (R = 0.99365), coupled with a low MRE of only 1.15621%, which underscores its excellent prediction precision. Wang et al. [18] implemented an ANN model to estimate the flow stress of P550 alloy under hot-working conditions. Additionally, they developed processing maps based on the predicted data. The GRU model, through its gating mechanism, effectively captures the dynamic temporal dependencies during the hot deformation process, exhibiting a balanced ability to handle long-term and short-term memory features in flow stress prediction. By its update gate and reset gate structure, this model can filter and retain critical thermodynamic information, significantly improving the accuracy of stress–strain modeling. Consequently, the GRU model demonstrates high prediction stability and generalization ability when describing physical processes with strong temporal correlations, such as hot deformation. He et al. [19] adopted a GRU model for the prediction of hot-compression behavior in 7046 aluminum alloy. Their results confirm that this approach offers remarkably accurate forecasts of flow stress during thermal deformation. However, as a model specifically tailored for the CoNiV MEA has not yet been established, existing models struggle to match its deformation behavior accurately. Therefore, leveraging the rapid development of modern computer technology, this study will establish ANN and GRU models to predict the behavior of this multi-element and nonlinear complex system.

These three types of models represent three mainstream modeling strategies, namely mechanism-based analysis, static data-driven modeling, and time-series deep learning, which collectively support the high-precision prediction of high-temperature flow stress of CoNiV MEA. This research will employ the Zerilli-Armstrong constitutive model, the ANN model, and the GRU model to establish constitutive models for the CoNiV MEA, analyze the model errors, and compare the characteristics of the different models.

## 2. Materials and Methods

Using vacuum induction melting technology under an argon atmosphere, high-purity (>99.95 wt.%) Co, Ni, and V elements (Yanbang new materials, Beijing Ryubon New Material Technology Co., Ltd., Beijing, China) were melted into an MEA ingot with a nominal composition of Co_33.33_Ni_33.33_V_33.34_ (at.%). Homogenization of the alloy ingot was ensured by applying four remelting steps. Following ASTM E209 requirements [20], cylindrical samples (Φ 8 mm × 12 mm) were fabricated by electrical discharge machining (EDM) from the middle section of the alloy ingot with dimensions of Φ 80 mm × 50 mm. The central position of every machined specimen was consistent with the geometric center of the ingot. Compression experiments were conducted on the CoNiV MEA using a Gleeble-3500 thermal simulator (Dynamic Systems Inc., Poestenkill, NY, USA) at deformation temperatures of 950, 1000, 1050, and 1100 °C and strain rates of 0.001, 0.01, 0.1, and 1 s^−1^. Based on CALPHAD phase diagram calculations, the CoNiV MEA exhibits a single-phase FCC structure between 900 and 1300 °C [11]. Accordingly, 1200 °C was selected as the heat treatment temperature. A heating rate of 10 °C/s raised the specimens to 1200 °C, followed by a 300 s hold to ensure uniform temperature throughout them. Subsequently, cooling at 5 °C/s brought the specimens to the set deformation temperature, where they remained for 10 s before undergoing hot compression treatment. After deformation to a true strain of 0.8, the specimens were rapidly water-quenched. To obtain reliable experimental results, each test was repeated three times in this work.

X-ray diffraction (XRD, Rigaku Smartlab SE, Rigaku Corporation, Tokyo, Japan) identified the phases of the original sample. Optical microscopy (OM, ZEISS optical microscope, Carl Zeiss AG, Oberkochen, Germany) and electron backscatter diffraction (EBSD, FEI Czech s.r.o., Brno, Czech Republic) served to characterize the microstructure of the alloy. To investigate the effect of the heat treatment (holding at 1200 °C for 300 s) on hot compression tests, specimens for OM and EBSD characterizations were cut from the heat-treated ingot using EDM. Specimens for OM observation were mechanically polished and subsequently etched in a mixed solution containing 75 vol.% HCl and 25 vol.% HNO_3_ (Zhengda Chemical Reagents, Tianjin Zhengda Chemical Reagent Co., Ltd., Tianjin, China). For EBSD testing, specimens were electropolished at 20 °C and 20 V in an alcohol-based solution with 10 vol.% perchloric acid.

R^2^, MAE, and RMSE serve as quantitative indicators to evaluate the predictive accuracy of the established model. The calculation formulas are as follows:

(1) $$R^{2}\;=\;\frac{{\left(\sum_{i=1}^{n}\left(A_{i}\;-\bar{\;A}\right)\left(M_{i}\;-\;\bar{M}\right)\right)}^{2}}{\sum_{i=1}^{n}{\left(A_{i}\;-\;\bar{A}\right)}^{2}{\left(M_{i}\;-\;\bar{M}\right)}^{2}}$$

(2) $$\mathrm{MAE}=\frac{1}{N}\sum_{i=1}^{N}\left|A_{i\;}-M_{i}\right|$$

(3) $$\mathrm{RMSE}=\sqrt{\frac{1}{N}\sum_{i=1}^{N}(A_{i}-{\;M}_{i}{)}^{2}}$$

In the formula, A represents the experimental flow stress, M refers to the predicted flow stress, $\overset{-}{A}$ represents the mean experimental flow stress, and N corresponds to the sample number of the training dataset.

## 3. Results and Discussion

### 3.1. Analysis of the Initial Microstructure and Mechanical Properties of CoNiV MEA

**Figure 1:**
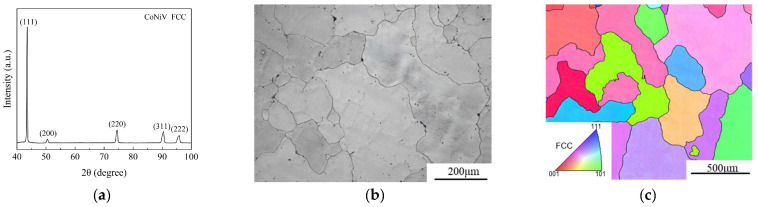
Initial microstructure of CoNiV MEA. (**a**) XRD pattern; (**b**) OM image; (**c**) IPF image.

XRD, OM, and EBSD were employed to characterize the microstructure of the CoNiV MEA before isothermal hot compression. Figure 1 depicts the microstructure of CoNiV alloy annealed at 1200 °C for 300 s. Before hot compression, the CoNiV MEA exhibits a single FCC structure and a primarily equiaxed grain morphology, with no pronounced crystallographic texture and an average grain size of 506.54 μm. It can be concluded that the heat treatment does not affect the deformation behavior during subsequent hot compression tests.

**Figure 2:**
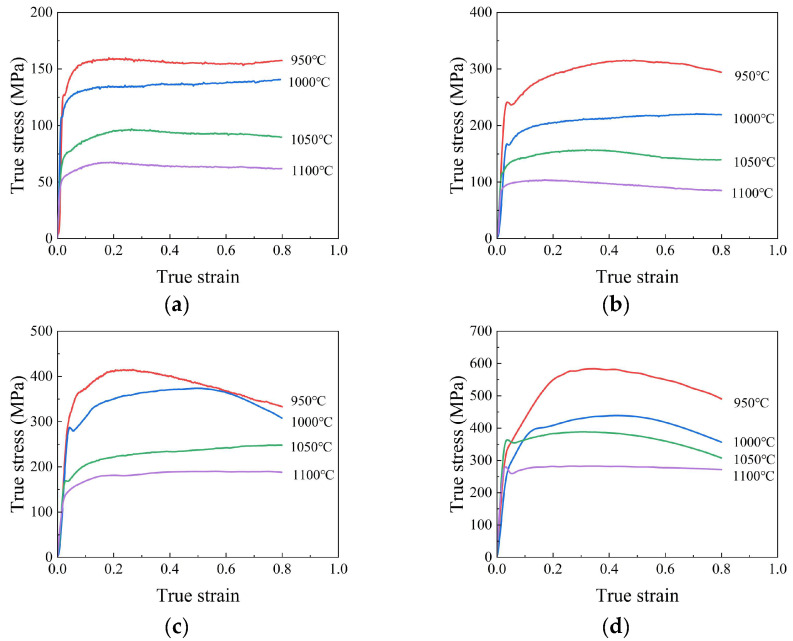
True stress-true strain curves of CoNiV MEA at different deformation conditions. (**a**) 0.001 s^−1^; (**b**) 0.01 s^−1^; (**c**) 0.1 s^−1^; (**d**) 1 s^−1^.

The true stress–strain curves of the CoNiV MEA under different temperatures (950–1100 °C) and strain rates (0.001–1 s^−1^) are presented in Figure 2. During the initial hot deformation stage, the flow stress increases with increasing strain, manifesting significant work-hardening behavior. Once the peak stress is attained, the flow curve enters a post-peak regime where the stress undergoes only marginal variations with increasing strain and eventually approaches a steady state. This phenomenon indicates that the deformation mechanism shifts from work-hardening dominance to a dynamic equilibrium between work hardening and dynamic softening. However, at lower temperatures and higher strain rates, a noticeable flow softening phenomenon can be observed [21]. After the flow stress reaches its peak value, dynamic recrystallization dominates the softening process and induces a continuous drop in flow stress [22]. At relatively high deformation temperatures and strain rates, the flow curve exhibits an initial peak followed by a substantial drop with increasing strain—a typical feature of discontinuous yielding behavior [23].

### 3.2. Establishment of Constitutive Model

#### 3.2.1. Zerilli-Armstrong (Z-A) Model

The principles of thermally activated dislocation mechanics underpin the Z-A model [24,25]. For FCC metals, this equation takes the following form:

(4) $$\sigma \;={\;C}_{0}\;+\;C_{2}\varepsilon^{0.5}\exp\left(-C_{3}T\;+\;C_{4}T\ln\overset{\cdot }{\varepsilon }\right)$$

In the formula, the units of C_0_ and C_2_ are MPa, while those of C_3_ and C_4_ are K^−1^. Using a variable n instead of the fixed value 0.5 improves the fitting results. At present, numerous modifications to the Z-A model enhance its prediction accuracy. Among them, the most widely applied improved model originates from the research by Samantaray et al. [26], and its expression is as follows:

(5) $$\sigma \;=\;\left(C_{1}\;+\;C_{2}\varepsilon^{n}\right)\exp\left[-\left(C_{3}\;+{\;C}_{4}\varepsilon \right)T^{*}\;+\;\left(C_{5}\;+\;C_{6}T^{*}\right){\ln\overset{\cdot }{\varepsilon }}^{*}\right]$$

In the formula, σ represents the stress, ε represents the strain, $T^{*}\;=\;T-T_{r}$, ${\overset{\cdot }{\varepsilon }}^{*}\;=\;\overset{\cdot }{\varepsilon }/{\overset{\cdot }{\varepsilon }}_{r}$, where ${\dot{\varepsilon }}_{r}$ and $T_{r}$ are the reference strain rate and reference temperature, and $\dot{\varepsilon }$ indicates the experimental strain rate. This equation includes more constants. A reference deformation temperature of 950 °C and a reference strain rate of 0.001 s^−1^ apply. Under the reference strain rate condition, $\ln\left(\dot{\varepsilon }\;/{\dot{\;\varepsilon }}_{r}\right)=0$, so Equation (5) can be simplified to Equation (6). Logarithmic transformation of both sides of Equation (6) produces Equation (7).

(6) $$\sigma =\left(C_{1\;}+{\;C}_{2}\varepsilon^{n}\right)\exp\left[-\left(C_{3}+{\;C}_{4}\varepsilon \right)T^{*}\right]$$

(7) $$\ln\sigma =\;\ln\left(C_{1\;}+C_{2}\varepsilon^{n}\right)\;-\left(C_{3}+C_{4}\varepsilon \right)T^{*}$$

**Figure 3:**
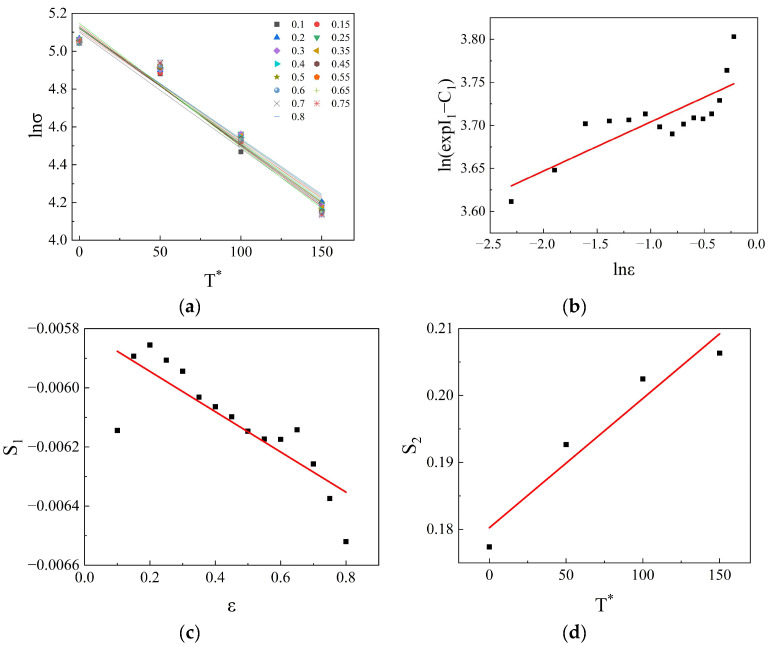
Formulation process of the Z-A constitutive equation. (**a**) $\ln\sigma -T^{*}$; (**b**) $\ln\left(\exp I_{1}-C_{1}\right)-\ln\varepsilon$; (**c**) $S_{1\;}-\varepsilon$; (**d**) $S_{2}-T^{*}$.

True stress–strain results from thermal simulation experiments provided data points at strain intervals of 0.05, ranging from 0.1 to 0.8. Linear fitting of lnσ against $T^{*}$ was performed using Equation (7), as shown in Figure 3a. Linear fitting produced the slope S_1_ and intercept I_1_, as presented respectively in Equations (8) and (9).

(8) $$I_{1}=\;\ln(C_{1\;}+C_{2}\varepsilon^{n})$$

(9) $$S_{1}=-\left(C_{3}+C_{4}\varepsilon \right)$$

Transforming Equation (8) yields Equation (10), where the value of C_1_ represents the yield stress at the reference temperature and reference strain rate. Based on the true stress and true strain data obtained from the experiments, C_1_ is determined to be 127.18 MPa.

(10) $$\ln\left(\exp I_{1}-{\;C}_{1}\right)=\ln C_{2}+n\ln\varepsilon$$

Applying Equation (10) to perform linear fitting yields the results presented in Figure 3b. The slope and intercept corresponding to n and C_2_, respectively, result in n = 0.05704 and C_2_ = 42.9927. Using Equation (9) to fit $S_{1}-\varepsilon$ yields the result presented in Figure 3c. The intercept equals—C_3_ and the slope equals—C_4_, giving C_3_ = 0.00581 and C_4_ = 0.00068.

Taking the natural logarithm of both sides of Equation (5) yields:

(11) $$\ln\sigma \;=\;\ln\left(C_{1}\;+{\;C}_{2}\varepsilon^{n}\right)\;-\;\left(C_{3}\;+\;C_{4}\varepsilon \right)T^{*}\;+\;\left(C_{5}\;+{\;C}_{6}\right)T^{*}{\ln\overset{\cdot }{\varepsilon }}^{*}$$

(12) $$S_{2}=C_{5}+{\;C}_{6}T^{*}$$

Substituting the previously determined values of C_1_, C_2_, C_3_, C_4_, and n into Equation (11). Under different temperature conditions, select data points at strain intervals of 0.05 from 0.1 to 0.8. Perform a linear fit of $\;\ln\sigma \;-\;{\ln\dot{\varepsilon }}^{*}f$, and identify the slope as S_2_. Use Equation (12) to linearly fit the average value of S_2_ against $T^{*}$, Figure 3d shows the results. The intercept and slope correspond to C_5_ and C_6_, yielding C_5_ = 0.18023 and C_6_ = 0.000193175. Thus, the finalized Z-A constitutive model for the CoNiV MEA takes the following form:

(13) $$\sigma \;=\;\left(127.18\;+\;42.9927\varepsilon^{0.05704}\right)\exp\left[-\left(0.00591\;+\;0.00068\varepsilon \right)T^{*}\;+\;\left(0.18023\;+\;0.000193175T^{*}\right){\ln\dot{\varepsilon }}^{*}\right]$$

**Figure 4:**
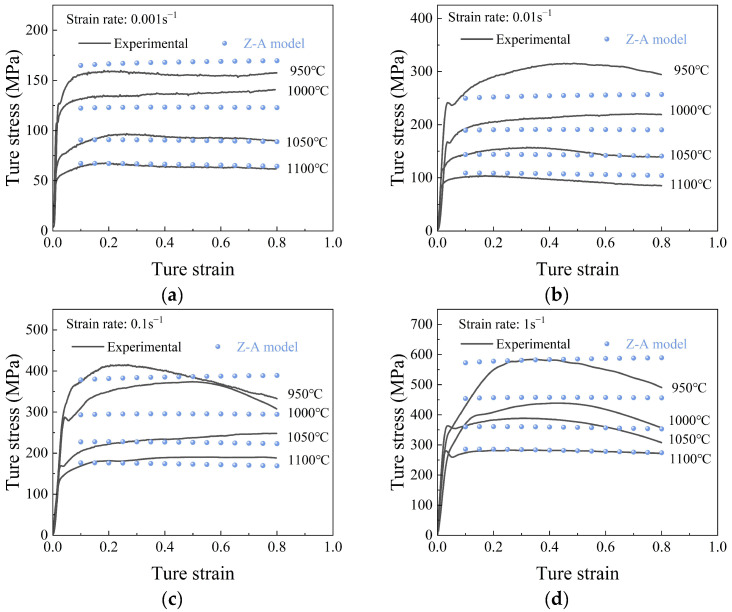
Comparison between the predictions of the conventional Z-A model and experimental values. (**a**) 0.001 s^−1^; (**b**) 0.01 s^−1^; (**c**) 0.1 s^−1^; (**d**) 1 s^−1^.

A higher R^2^ value (near 1) and lower MAE and RMSE values (close to 0) signal better predictive performance of a model [27]. The Z-A model delivers an R^2^ of 0.71983, an MAE of 21.0134, and an RMSE of 123.255. The Z-A model is commonly used to capture the strain hardening and dynamic recovery behavior of metallic materials at elevated temperatures. The comparison of the stress–strain curves in Figure 4a–d shows that the model aligns well with the experimental curves at lower strain rates (0.001 s^−1^), as well as at all strain rates under the temperatures of 1050 °C and 1100 °C. However, certain deviations occur at higher strain rates (0.1 s^−1^ and 1 s^−1^), particularly at lower temperatures. These discrepancies expose the limited predictive accuracy of the Z-A model and its inability to describe hot deformation behavior over the whole strain range. This is likely because the model does not adequately describe the more pronounced dynamic recrystallization or dislocation evolution mechanisms under high-strain-rate conditions [28].

**Figure 5:**
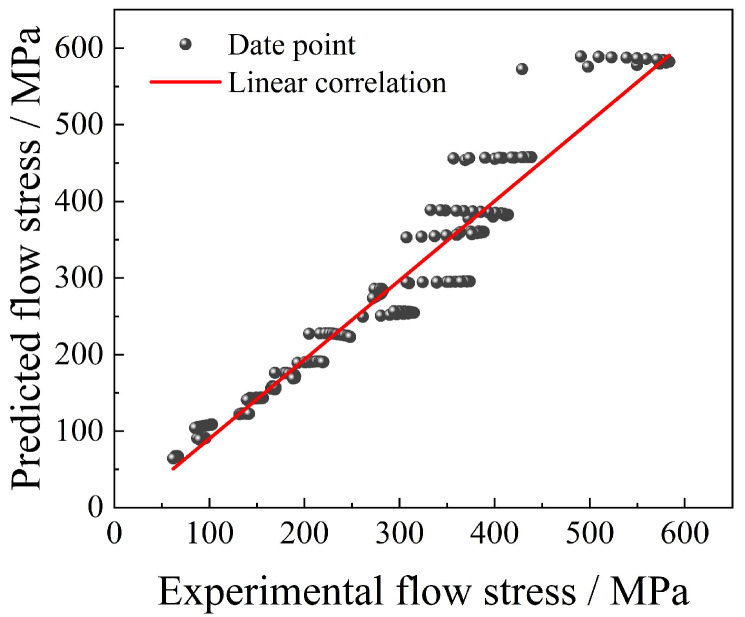
Linear fitting effectiveness between the predictions of the Z-A model and experimental values.

Plotting the linear regression of experimental flow stress against constitutive equation predictions and adopting the calculated adjusted R^2^ was used to verify the predictive reliability of the Z-A model. Figure 5 plots the linear fitting relationship between the experimentally obtained flow stress and the flow stress forecasted by the constitutive model. Numerical results show that the adjusted R^2^ of the Z-A constitutive equation reaches 0.95336.

The traditional Z-A model has a high parameter count, which leads to computational complexity and lengthy solution times [29,30]. Thus, accurate simulation of stress–strain curves demands models that can manage the complex datasets encountered in thermal processing. Unlike the Z-A model, machine learning (ML) approaches directly acquire the desired output from experimental data, independent of conventional mathematical formulations or physical principles. Hence, ML models offer the dual benefits of reduced computational expense and enhanced precision in characterizing alloy rheological behavior.

#### 3.2.2. Artificial Neural Network (ANN) Model

As a computational model, the ANN model draws its inspiration from the structure and operation of biological neural systems [31]. Its core principle is to simulate complex functional mappings using a hierarchy of interconnected simple processing units called neurons. In each neuron, weighted inputs coming from the previous layer are first summed, after which a nonlinear activation function is applied to produce the output. This nonlinear property gives the network the capability to approximate any continuous function of arbitrary complexity. The network computes predicted values through forward propagation and iteratively updates its internal parameters via back-propagation during training. Equation (14) to Equation (16) present the relevant processes [32]. Using the discrepancy between the predicted output and the true data, gradients are calculated layer by layer, after which the weight and bias parameters of each connection are updated. In this data-driven manner, the model uses optimization methods such as gradient descent to automatically minimize the error and progressively extracts distributed feature representations—from low-level to high-level—from the data layer by layer.

(14) $$z_{j}=\phi \left(\sum_{i=1}^{N}w_{\mathrm{ij}}^{\left(1\right)}x_{i}+b_{j}^{\left(1\right)}\right)$$

(15) $$y_{k}=\phi (\sum_{j=1}^{M}w_{\mathrm{kj}}^{\left(2\right)}z_{j}+{\;b}_{k}^{\left(2\right)})$$

(16) $${\hat{y}}_{k}=g(y_{k})$$

**Figure 6:**
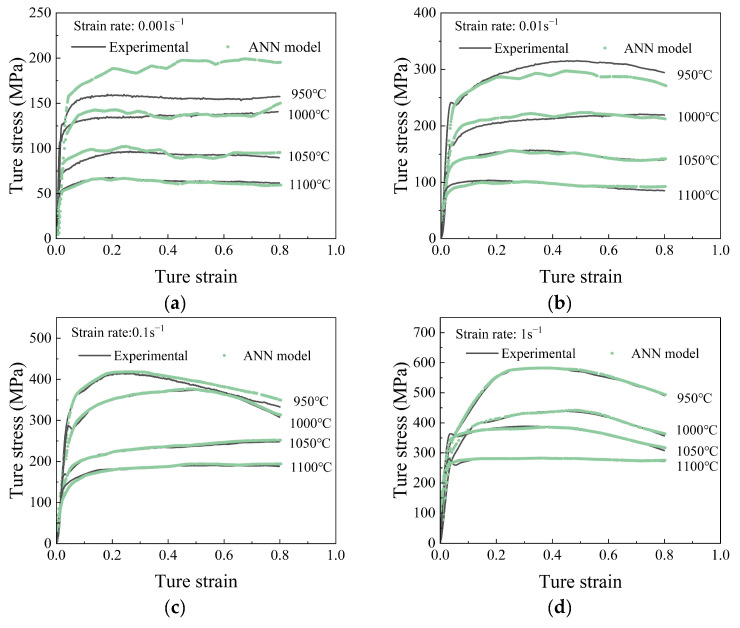
Comparison between the predicted values of the ANN model and the experimental values. (**a**) 0.001 s^−1^; (**b**) 0.01 s^−1^; (**c**) 0.1 s^−1^; (**d**) 1 s^−1^.

Figure 6 plots the experimentally acquired measurements against the predictive outputs of the ANN model. Compared with the Z-A model, the ANN model delivers remarkably superior predictive performance, as clearly reflected in the figure. The ANN prediction curve closely matches the experimental curve in overall trend, especially in the transition region between strain hardening and dynamic softening stages. However, under low-strain-rate and low-temperature conditions, a noticeable deviation persists between the two curves. The ANN is a powerful interpolation tool, but its performance degrades when extrapolating beyond the training data range. This region lies at the edge of the parameter space in the training dataset, and uncertainty increases when the model extrapolates in this region.

#### 3.2.3. Gated Recurrent Unit (GRU) Model

Cho et al. [33] proposed GRU as an important variant of the recurrent neural network (RNN). It aims to address gradient instability in traditional RNNs and simplify the structure of the long short-term memory (LSTM) network. Unlike traditional LSTM networks, the GRU simplifies internal gate structures by merging the input gate and forget gate into an individual update gate. This architecture consists of two gates in total, and the update gate is responsible for determining the reserved proportion of historical data inside the current state. In contrast, the reset gate controls how much historical information to forget when producing a new candidate state. The GRU performs a linear transformation on the current input and the previous hidden state, applies the sigmoid function to generate gating signals, and then progressively updates the hidden state by combining the candidate hidden state with the gating values. This design preserves long-term dependencies while reducing the number of parameters, making training more efficient.

**Figure 7:**
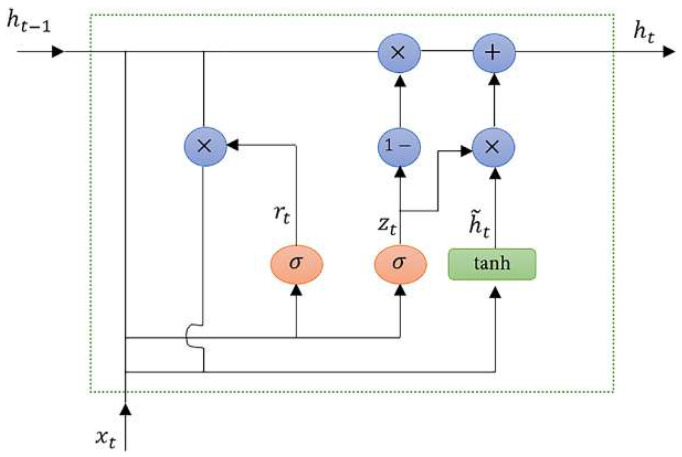
Schematic diagrams of the ANN model [34].

Figure 7 displays the schematic diagram of the GRU model [34]. The following expression describes the fundamental procedures of the GRU [35]:

(17) $$z_{t}\;=\;\sigma (W_{\mathrm{xz}}x_{t}\;+{\;W}_{\mathrm{hz}}h_{t-1}\;+{\;b}_{z})$$

(18) $$r_{t}=\sigma (W_{\mathrm{xr}}x_{t}+W_{\mathrm{hr}}h_{t-1}+b_{r})$$

(19) $${\tilde{h}}_{t}=\tanh(W_{\mathrm{xh}}x_{t}+r_{t}\;⊙{\;W}_{\mathrm{hh}}h_{t-1}+{\;b}_{h})$$

(20) $$h_{t}=(1-z_{t}⊙{\;h}_{t-1})+z_{t}⊙\;{\tilde{h}}_{t}$$

In the formula, $z_{t}$ represents the update gate, $r_{t}$ represents the reset gate, ${\tilde{h}}_{t}$ is the new candidate state, $h_{t}$ is the hidden state at time t, σ denotes the sigmoid function, W corresponds to the weight matrices, and b represents the bias terms.

**Figure 8:**
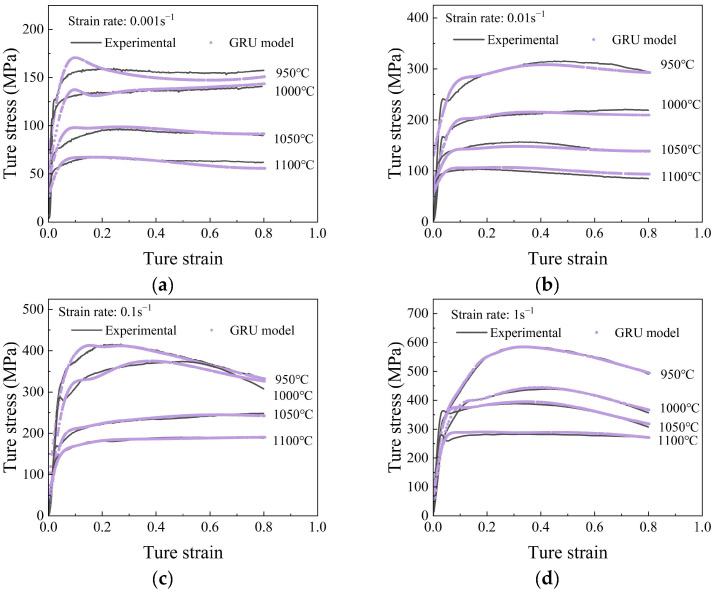
Comparison between the predicted values of the GRU model and the experimental values. (**a**) 0.001 s^−1^; (**b**) 0.01 s^−1^; (**c**) 0.1 s^−1^; (**d**) 1 s^−1^.

Figure 8 presents the true stress–true strain curves of the alloy under different deformation conditions and a comparison between the experimental results and the GRU model predictions. The curves predicted via the GRU model are highly consistent with those obtained from hot compression experiments. Such good agreement proves that the constructed model can well reflect the flow properties of the alloy under various hot deformation circumstances, thereby exhibiting reliable and superior prediction capacity. Through its update gate and reset gate mechanisms, the GRU can better model this path-dependent mechanical response, thereby achieving smoother predictions that are closer to the experimental curves in terms of evolutionary trends. This may account for its slightly lower errors than the ANN model at certain strain stages.

### 3.3. Predictability Comparison

To ensure that machine learning models demonstrate excellent predictive capabilities in both controlled experimental settings and unknown scenarios, it is essential to ensure that the dataset is sufficiently representative and covers crucial influencing factors as well as potential operational variations. The 6800 groups of data acquired via hot compression experiments were employed to train the machine learning model. To train and verify the established model, all thermal simulation compression test data were randomly allocated to three non-overlapping subsets. The allocation proportion was set to 70% for model training, 20% for parameter validation, and the final 10% for generalization testing. Three physical quantities, including the logarithm of strain rate, deformation temperature, and strain, were chosen as input variables, and flow stress was regarded as the output target of the prediction model. All data were further adjusted and scaled via data standardization [36]. The formula for standardizing the data is as follows [37]:

(21) $$X^{’}\;=\;\frac{X\;-\overset{-}{X}}{\sigma_{x}}$$

In the formula, X refers to the raw experimental data, $\overset{-}{X}$ denotes the mean value of the experimental data, $\sigma_{X}$ stands for the standard deviation, and $X^{’}$ represents the standardized result of the raw data. In addition to data standardization, applying ten-fold cross-validation to the training data in each iteration helps to prevent the ML model from overfitting. Repeated iterations and ongoing hyperparameter tuning finally yielded the optimal predictive model. The hyperparameters of the established ANN and GRU models are summarized below. The ANN has two hidden layers with 128 and 64 neurons using the ReLU activation function. The GRU is equipped with two hidden layers with 50 neurons per layer, adopting Tanh and Sigmoid as the activation and recurrent activation functions, respectively.

**Figure 9:**
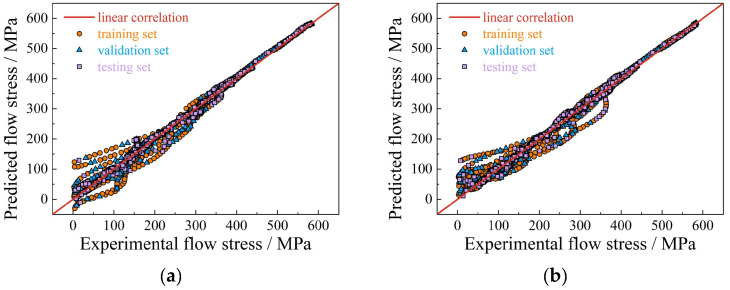
The correlation between the experimental results and the predicted results of different ML models. (**a**) ANN; (**b**) GRU.

Two machine learning models, ANN and GRU, were employed to predict and analyze the experimental stress–strain data. Figure 9 compares the experimental flow stress with the predictions from two different ML models. In Figure 9b, the data from the GRU model lie closer to the asymptotic line, indicating higher forecasting accuracy. The GRU model demonstrates superior predictive accuracy and generalization relative to its ANN counterpart, as the latter’s predictions exhibit considerably larger discrepancies from the experimental observations.

The R^2^ values for the ANN model and the GRU model are 0.98974 and 0.98981. The GRU model outperforms the ANN model in terms of R^2^. However, overfitting can also lead to the R^2^ value being close to 1, while the model exhibits persistently low generalization performance. Therefore, it is necessary to evaluate further the predictability and generalization ability of the model using MAE and RMSE. The computed MAE for the ANN model is 7.23299, and for the GRU model is 6.29621; the respective RMSE values are 13.15198 and 13.10832, indicating that the GRU model outperforms the ANN model in terms of prediction accuracy and generalization ability. This is consistent with the results of the analysis in Figure 8. Therefore, the GRU model holds the most promise for accurately predicting the flow stress of the CoNiV MEA.

Machine learning offers significant advantages in predicting flow stress; it can accurately fit nonlinear thermal deformation behavior, effectively reduce experimental costs, and is easily integrated with finite element simulations. However, such data-driven models lack physical interpretability, which limits their widespread industrial application. In future research, embedding the physical mechanisms of hot deformation into the ML model endows it with extrapolation capabilities while maintaining high prediction accuracy, thereby promoting its industrial applications.

## 4. Conclusions

(1) Within the hot deformation temperature range of 950–1100 °C and strain rate range of 0.001–1 s^−1^, the flow stress of the CoNiV medium-entropy alloy exhibits an initial rising trend with the increase in strain and subsequently tends to reach a stable state. Deformation temperature and strain rate impose remarkable influences on the hot flow characteristics of the alloy.
(2) Although the Zerilli–Armstrong constitutive model, based on thermally activated dislocation theory, yields reasonably accurate predictions at low strain rates, its accuracy deteriorates markedly at high strain rates and in the low-temperature regime. As a result, it fails to capture the rheological behavior over the entire processing window. The low predictive accuracy is further verified by statistical evaluations, with R^2^ = 0.71983, MAE = 21.0134, and RMSE = 123.255, demonstrating that its overall predictive performance is insufficient for reliable flow stress prediction.
(3) Among the two machine learning models, the GRU model achieved the highest prediction accuracy due to its ability to capture temporal dependencies through its gated recurrent mechanism, with an R^2^ value of 0.98981, an MAE of 6.29621, and an RMSE of 13.10832. The GRU model thus outperforms the ANN model, which had an R^2^ of 0.98974, MAE = 7.23299, and RMSE = 13.15198. The GRU model can thus provide reliable support for the precise formulation and optimization of hot working process parameters for CoNiV MEA.

## Data Availability

The original contributions presented in this study are included in the article. Further inquiries can be directed to the corresponding author.
